# The Impact of Preoperative Radiotherapy and Chemotherapy on Autologous Breast Reconstruction Outcomes—A Retrospective Single-Center Study

**DOI:** 10.3390/cancers17030512

**Published:** 2025-02-04

**Authors:** Caterina M. Nava, Jérôme Martineau, Edward T. C. Dong, Gauthier Zinner, Carlo M. Oranges

**Affiliations:** Department of Plastic, Reconstructive, and Aesthetic Surgery, Geneva University Hospitals, Geneva University, 1205 Geneva, Switzerland; caterinamaria.nava@gmail.com (C.M.N.); jerome.martineau@hug.ch (J.M.); edward.dong@etu.unige.ch (E.T.C.D.); gauthier.zinner@hug.ch (G.Z.)

**Keywords:** radiotherapy, chemotherapy, autologous free flap, DIEP, microvascular

## Abstract

Background: While radiotherapy (RT) and chemotherapy (CT) significantly improve breast cancer outcomes, they may affect breast reconstruction by causing vascular damage and delayed wound healing. This retrospective study evaluates how preoperative RT, CT, or the combination of both impacts intraoperative and postoperative outcomes in immediate or delayed deep inferior epigastric perforator (DIEP) flap breast reconstructions. Methods: We conducted a single-center review of all patients undergoing autologous DIEP flap reconstruction after mastectomy between 2018 and 2024. Patients were divided into four groups: RT only, CT only, a combination of RT and CT, and a control group with no preoperative therapies. Intraoperative and postoperative outcomes were then compared among these groups, with statistical significance defined as *p* < 0.05. Results: We included 114 patients representing 141 DIEP-flap breast reconstructions. Flap survival rate was 98.5%. In the univariate analysis, total microvascular recipient site complications were significantly higher in the RT + CT group (14.0%, *p* = 0.021). Donor-site complication rates differed significantly among the four groups (*p* = 0.025), with the highest rate observed in the RT + CT group (44.7%). In the logistic regression analysis, ischemia time was found as an independent risk factor for total recipient site complications, but not for microvascular complications (OR = 1.019, 95%-CI = 1.004–1.035, *p* = 0.014). Conclusions: Combined RT + CT significantly increased microsurgical complications. Ischemia time correlated with higher odds of total recipient site complications. Individualized patient management and diminished ischemia time are likely to improve flap survival.

## Simple Summary

Breast cancer treatments often involve surgery, chemotherapy, and radiotherapy to control the disease and improve survival. However, these treatments can affect the success of breast reconstruction procedures, particularly those using autologous tissue such as the deep inferior epigastric perforator (DIEP) flap technique. Some articles have addressed this issue, but the results remain heterogeneous. This study compares the outcomes of DIEP flap reconstructions in breast cancer patients who received chemotherapy, radiotherapy, or both prior to the reconstruction, against those who did not receive any of these treatments. By analyzing complications and surgical outcomes, this retrospective study aims to guide surgeons in managing patients with complex treatment histories. Understanding these effects will help improve patient care and outcomes in breast cancer reconstruction.

## 1. Introduction

Breast cancer remains the most common malignancy among women worldwide, necessitating a multimodal approach to treatment including surgery, radiotherapy (RT), and chemotherapy (CT) [1,2]. RT is critical for reducing locoregional recurrence and improving overall survival rates both in patients undergoing breast-conserving surgery and mastectomy [3,4,5,6]. Similarly, CT aims to downstage the primary tumor and axillary lymph nodes, increasing the rates of pathologic complete response and reducing the risk of distant recurrence [7,8,9,10]. A curative approach that can combine oncological treatment and breast reconstruction yielding a pleasing and aesthetic breast, such as the deep inferior epigastric artery perforator (DIEP) flap, among others, is therefore often required [11,12,13,14].

Laboratory research has demonstrated that radiation increases local collagen deposition, disrupts angiogenesis in vascular beds, and heightens fibrosis in human tissues [15]. Combined with other in vivo changes such as edema and contracture, preoperative RT may compromise the success of microvascular procedures [16,17,18]. Similarly, CT has cytotoxic effects on the endothelium of small vessels and the immune system, potentially increasing the risk of complications, including delayed wound healing and infections [19].

Many studies have examined the impact of prior RT or CT on complication rates in breast cancer patients undergoing delayed autologous breast reconstruction [20,21,22,23,24,25,26]. For instance, a recent review by Ward et al. found rates of surgical complications and reconstructive outcomes within normal limits among patients with preoperative chemoradiotherapy undergoing mostly DIEP flap reconstructions [27]. Thiruchelvam et al. in a prospective study including women with preoperative RT and CT showed a low rate of open wounds, mastectomy skin necrosis, fat necrosis, and unplanned returns to the operating theater with no DIEP flap failures [28].

However, the literature remains inconsistent, and no study to date has specifically examined the combined effects of both preoperative RT and CT on DIEP flap outcomes compared to patients with RT- and CT-naïve recipient sites.

This retrospective study aims to evaluate the impact of preoperative oncological treatments—RT alone, CT alone, and the combination of RT and CT—on intraoperative and postoperative outcomes in patients undergoing DIEP flap breast reconstruction. The outcomes in these groups will be compared to a control group of patients who did not receive preoperative RT or CT.

## 2. Materials and Methods

In this single-center study, medical charts of female patients who underwent a DIEP flap breast reconstruction between January 2018 and June 2024 were retrospectively reviewed. This study received approval from the relevant ethics committee (Project ID: CCER 2024-01344) and adheres to STROBE guidelines.

Patients were included if they had a mastectomy, followed by an immediate or delayed DIEP flap breast reconstruction. Patients were excluded if RT or CT was administered after breast reconstruction.

**Figure 1:**
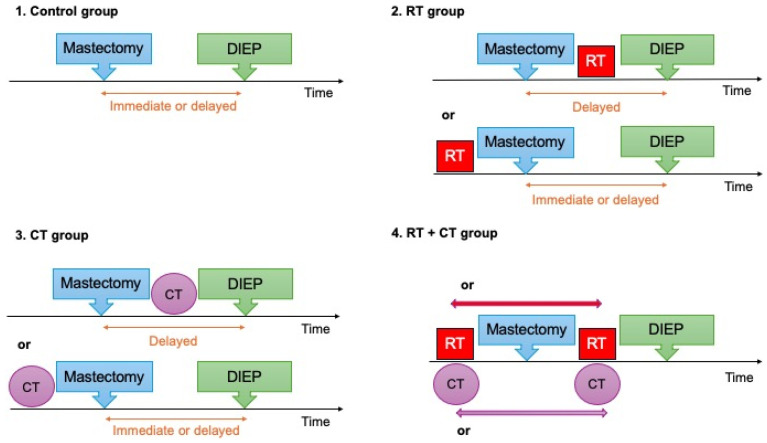
Study groups based on preoperative treatment.

Patients were categorized into four groups: a control group with no preoperative RT or CT and three treatment groups based on their exposure to RT only, CT only, or the combination of both CT and RT prior to DIEP flap breast reconstruction (Figure 1).

Preoperative RT included any radiation administered to the chest wall and axilla region on the side of the reconstruction. Indications for RT included prior breast-conserving therapy and postmastectomy RT prior to delayed reconstruction.

Preoperative CT included any chemotherapy given before DIEP flap reconstruction, typically administered prior to mastectomy but occasionally after mastectomy in delayed reconstructions. 

All surgeries were performed by the microsurgical team of an academic institution, which always included at least one experienced microsurgeon.

Both bilateral and unilateral reconstructions were assessed. In the bilateral cases, each flap was analyzed as a separate data point in postoperative complications, and each breast of the same patient was classified in the study group corresponding to the prior treatment received (RT, CT, RT + CT, or none). Demographic data such as age, body mass index (BMI), active smoking status, hypertension (HTN), diabetes, American Society of Anesthesiologists (ASA) scores, and history of previous abdominal surgery were recorded on a per-patient basis. Intraoperative and postoperative outcomes were also collected on a per-flap basis for the recipient site and on a per-patient basis for the donor site.

To assess complications based on retrospective data, the reported complications were defined as follows:

1. Seroma: a collection of serous fluid in the surgical bed detected by imaging and necessitating needle aspiration or surgical reintervention.
2. Hematoma: a localized collection of blood within the surgical space, typically requiring drainage or intervention if significant.
3. Wound infection: Clinical or laboratory evidence of infection—such as purulent drainage, positive microbial cultures, and/or local/systemic signs of infection (redness, warmth, fever).
4. Wound dehiscence: A mechanical separation of the layers of a previously approximated wound (partial or complete), often necessitating additional intervention (negative pressure wound therapy (NPWT), re-suturing, local flap)
5. Delayed wound healing: A wound that fails to progress through normal phases of healing within the expected postoperative timeframe, without mechanical separation of wound edges (i.e., no dehiscence), and with no other surgical intervention than simple dressings and wound care with topical agents. These cases benefited from ambulatory follow-up at our wound care center.
6. A “microvascular complication” was defined as any compromise in flap perfusion necessitating urgent surgical re-exploration, whether or not the arterial or venous anastomoses required revision.

### Statistical Analysis

Once collected, the data were imported into SPSS version 29.0.2.0 for statistical analysis (I.B.M., Armonk, NY, USA).

Continuous variables were reported as mean ± standard deviation (SD), while categorical variables were summarized as frequencies and percentages. ANOVA was used to compare continuous variables across the four groups, and post-hoc analyses were performed when necessary. Chi-square tests were applied to compare categorical variables, including complication rates between the groups. When expected cell frequencies were 5 or fewer, Fisher’s exact test was used instead.

To adjust for potential confounders and to assess the independent effects of RT, CT, and the combination of RT and CT on intraoperative and postoperative outcomes, binary logistic regression analysis was performed. Covariates that showed significant differences between groups or had clinical relevance to total recipient site, microvascular recipient site, or donor site complications were included in the models. A *p*-value of 0.05 or less was considered statistically significant.

## 3. Results

### 3.1. Patients’ Characteristics

A total of 114 patients were included, representing a total of 141 DIEP flap breast reconstructions: 29 in the control group (25.4%), 21 in the RT group (18.4%), 17 in the CT group (14.9%), and 47 in the combined RT + CT group (41.2%) (Table 1). The RT group had a significantly higher mean age (54.3 ± 11.1 years) compared to the others (*p* = 0.037). Active smoker status was the highest in the control group (31.0%), followed by the CT (23.5%), RT + CT (21.3%), and RT groups (19%) (*p* = 0.05). No significant differences were found in other baseline characteristics, such as BMI, ASA score, HTN, diabetes, and abdominal surgical history across groups.

### 3.2. Operative Variables

Significant intraoperative differences were observed among groups (Table 2). Immediate reconstructions were more common in the control group (41.4%) compared to the RT (23.8%), CT (41.2%), and combined RT + CT (14.9%) groups (*p* = 0.04). Operative time was longer in the CT group (601 ± 144.4 min) compared to combined RT + CT (506.9 ± 107 min), RT (486.6 ± 133.6 min), and the control group (466 ± 127.1 min) (*p* = 0.005), but the CT group also had a higher proportion of bilateral reconstructions (70.5%, *p*< 0.001). Excluding bilateral cases, operative times for unilateral reconstructions were similar across groups: 472.67 ± 69.51 min (CT), 471.1 ± 77.37 min (combined RT + CT), 468.63 ± 88.69 min (control group), and 442.29 ± 96.30 min (RT) (*p* = 0.681). Oncologic mastectomies were more common in the RT and combined RT + CT groups (100%), followed by the control group (93.9%) and CT group (72.4%), while prophylactic mastectomies were more common in the CT (27.5%), followed by the control group (6.1%) (*p* < 0.001). Nipple-sparing mastectomy (NSM) was more common in the CT group (37.9%, *p* = 0.043), while simple mastectomies were more frequent in the combined RT + CT group (29.8%, *p* = 0.004). Skin-sparing mastectomies (SSM) were equally distributed among groups (*p* = 0.309). Other factors, including length of hospital stay, number of perforators, target vessels, venous anastomosis with coupler, and ischemia time, did not differ significantly between groups. Patients received RT either after previous breast-conserving surgery and before mastectomy for recurrence (47.6% in RT, 36.2% in RT + CT) or after mastectomy and prior to delayed reconstruction (52.4% in RT, 63.8% in RT + CT groups). There was no statistically significant difference between groups regarding the timing of RT.

### 3.3. Radiotherapy and Chemotherapy Characteristics:

The mean radiotherapy doses in Grey (Gy) were similar between the RT group (50.5 ± 10.8) and combined RT + CT group (53.0 ± 5.3) (*p* = 0.190). The intervals between completion of RT and DIEP flap breast reconstruction were 69.2 (±61.1) and 49.2 (±58.9) months in the RT and combined RT + CT groups, respectively. The intervals from chemotherapy completion to reconstruction were 37.2 (±59.0) and 49.1 (±59.1) months in the CT and RT + CT groups, respectively, with no significant difference (*p* = 0.390).

### 3.4. Complications

In this study, complications were categorized into two main groups: recipient site complications (Table 3) and donor site complications (Table 4).

The overall recipient site complication rate was highest in the combined RT + CT group (24.6%), followed by the RT group (18.2%), the control group (12.1%), and the CT group (10.3%) (Table 3). The trend suggests a higher complication rate in the combined RT + CT group, but this difference was not statistically significant (*p* = 0.306). Seroma occurred in one RT patient (*p* = 0.141) and needed surgical evacuation. Hematoma rates were the highest in the RT group (4.5%) but were not significantly different among groups (*p* = 0.333); all five patients underwent a surgical evacuation. Wound infections occurred in one CT and one combined RT + CT patient (*p* = 0.638) and needed oral antibiotic treatment. Wound dehiscence rates were similar across all groups (*p* = 0.993), with one RT + CT patient requiring post-operative NPWT, three requiring minor surgical revision of the wound edges and re-suturing, and one RT patient needing a local flap for coverage. Delayed wound healing and partial nipple-areolar-complex (NAC) necrosis were infrequent, with no significant differences observed. Overall, no significant differences were found among groups for non-microvascular recipient site complications.

However, the total microvascular complication rate was the highest in the combined RT + CT group (14.0%) and was statistically different from the rates observed in the control group (3.0%) and the RT and CT groups (both 0.0%) (*p* = 0.021) (Table 3). Venous congestion occurred in one patient (3%) in the control group, with no cases in the other groups (*p* = 0.348); the flap was revised, and venous kinking was resolved. Venous thrombosis was observed in two patients postoperatively (3.5%) in the combined RT + CT group and 0.0% in the other groups, both of these cases requiring reoperation for venous anastomosis revision (Table 3). One arterial thrombosis occurred intraoperatively in the combined RT + CT group (1.8%), requiring intraoperative revision of the arterial anastomosis before closure. Notably, there were two cases of total flap loss (3.5%) in the combined RT + CT group compared to none in the other groups (*p* = 0.393). Partial flap loss was recorded in three patients (5.3%) in the combined RT + CT group compared to none in the other groups (*p* = 0.211) (Table 3). Three of these five compromised flaps required a postoperative arterial anastomosis revision, while two cases of partial flap necrosis returned to the operating room for surgical flap debridement without the need for anastomosis revision. When analyzed individually, none of these microvascular recipient site complications showed statistically significant differences between groups.

Abdominal complication rates differed significantly among the groups (*p* = 0.025), with the highest rate in the combined RT + CT group (44.7%), followed by the CT group (35.3%), the control group (24.1%), and the RT group (9.5%) (Table 4). Other cosmetic complications at the donor site included hypertrophic scars (6.9% in the control group, 4.8% in the RT group, 17.6% in the CT group, and 0% in the RT + CT group, *p* = 0.046) and dog-ear scar deformities (0% in the control group, 4.8% in the RT group, 0% in the CT group, and 2.1% in the RT + CT group, *p* = 0.580). These were not counted in the total donor-site complication rates.

### 3.5. Logistic Regression Model

We selected variables for binary logistic regression based on statistical significance in univariate analyses and clinical relevance to complications. In the total recipient site complications model, among the variables studied, ischemia time emerged as a significant predictor (*p* = 0.014), with an odds ratio (OR) of 1.019 (95% confidence interval (CI) 1.004–1.035).

In contrast, other variables such as age (OR = 1.023, 95%-CI: 0.960–1.090, *p* = 0.475), smoking status (OR = 1.696, 95%-CI: 0.539–5.33, *p* = 0.366), radiotherapy (RT) (OR = 1.302, 95%-CI: 0.236–7.191, *p* = 0.762), chemotherapy (CT) (OR = 0.832, 95%-CI: 0.162–4.277, *p* = 0.826), combined RT and CT (OR = 1.607, 95%-CI: 0.456–5.659, *p* = 0.460), and operation time (OR = 0.999, 95%-CI: 0.995–1.003, *p* = 0.717) did not show statistically significant associations with recipient site complications (Table 5).

In the model for total microvascular recipient site complications (Table 6) and donor-site complications (Table 7), no independent risk factors were identified.

## 4. Discussion

In this retrospective study of 114 patients undergoing 141 DIEP flap breast reconstructions, we examined the impact of preoperative RT and/or CT on surgical outcomes by comparing them with a control group that did not receive CT or RT prior to DIEP flap breast reconstruction. We found that patients who received both RT and CT prior to reconstruction had a significantly higher rate of microvascular recipient site complications compared to those who received either treatment alone or no treatment at all. However, this did not affect the flap loss rate significantly. Conversely, the total recipient site complications rate (including non-microvascular) was not significantly different among groups. Ischemia time was identified as an independent risk factor for total recipient site complications, but not for microvascular recipient site complications, in the logistic regression models.

We focused on analyzing outcomes that reflect the overall success of flap surgeries. Postoperative complications are directly linked to surgical success, while factors such as the duration of the surgery and ischemia time serve as indirect indicators of potential difficulties encountered during reconstruction and microsurgical anastomosis.

Our findings indicate that the combination of RT and CT increases microvascular recipient site complications compared to administering just one treatment or none. While previous research has examined the influence of RT and CT individually, to our knowledge, no studies have specifically focused on the combined effect of preoperative RT and CT administration before a DIEP flap reconstruction.

Fundamental research studies have demonstrated that radiation led to an increase in local collagen deposition, a disruption of angiogenesis in vascular beds, and a heightened fibrosis in human tissues. These factors could reduce perfusion and cause more extensive parenchymal changes in reconstructed breasts [15]. A recent study by Miyazawa et al. [29] compared irradiated and non-irradiated patients undergoing free flap breast reconstruction—regardless of whether they had received preoperative chemotherapy—and found no significant differences in the rates of vascular complications or reoperation. Similarly, other studies reported no differences in microvascular and wound complication rates between previously irradiated versus non-irradiated breasts, regardless of the previous chemotherapy status [30,31].

Conversely, some studies have indicated that RT increases the rate of vascular complications, the majority of which appear intraoperatively, without hindering the overall success of reconstruction and without affecting any postoperative complications (postoperative thrombosis, flap loss, flap necrosis, fat necrosis, hematoma, seroma, or delayed wound healing) [32,33].

Studies on previously CT-treated patients showed no significant differences in microvascular, recipient site, and donor-site complication rates [34,35,36,37,38]. Interestingly, in our study, the total donor-site complication rates differed significantly among the groups, with the highest rate observed in the RT + CT group. This result cannot be attributed to demographics or associated comorbidities within the RT + CT group. As RT was exclusively administered to the recipient site, it is reasonable to infer that it does not directly influence the donor-site area.

Moreover, experimental studies have shown that CT impairs cell division, inhibits cellular metabolism and angiogenesis, and interferes with several molecular pathways [39]. Taken together, these mechanisms could affect tissue quality and result in poorer scar healing outcomes, explaining our results.

Our study also proves that each additional unit increase in ischemia time was associated with a 1.9% increase in the odds of experiencing total complications at the recipient site. Although we have shown an association between ischemia time and total recipient site complications, this was not confirmed in the binary logistic regression analysis for microvascular recipient site complications alone. A similar result has already been reported in a recent large meta-analysis including 5636 patients and 6884 free flaps, demonstrating that cases with ischemia time exceeding 60 min were associated with a heightened risk of total recipient site complications, complete and partial flap loss, hematoma, and infection [40]. The larger number of flaps analyzed in this meta-analysis allows for more robust and reliable conclusions. Two other studies that have addressed microvascular complications according to ischemia time conclude that flaps with ischemia longer than 99.5 min and 1.5–2 h are at higher risk of developing fat necrosis and microvascular complications, respectively [41,42].

Similar to other free tissue transfers, the DIEP flap is vulnerable to ischemia-reperfusion injury, which may compromise its viability. During primary ischemia, cells switch to anaerobic metabolism, resulting in endothelial damage, interstitial edema, and inflammatory responses [43]. Upon reperfusion, neutrophil influx exacerbates oxidative injury, influencing whether the tissue survives or experiences a no-reflow phenomenon [44].

Hyperbaric oxygen therapy (HBOT) has been studied as a prophylactic treatment for previously irradiated patients to facilitate the flap in setting by improving vascularity, cellularity, and collagen deposition [45]. In our study, we used HBOT in selected patients with threatened flaps during the early postoperative recovery period. The selection of therapy was at the discretion of the senior author, highlighting a potential bias in the final flap complication rates.

In our study, only autologous breast reconstructions performed with DIEP flaps were included. There are numerous procedures available for autologous breast reconstruction nowadays; however, the DIEP flap continues to be considered the gold standard [46,47,48,49]. Alternatives include thigh-based flaps such as the profunda artery perforator (PAP) and the transverse upper gracilis (TUG), buttock-based flaps such as the inferior gluteal artery perforator (IGAP) and the superior gluteal artery perforator (SGAP), and the latissimus dorsi (LD) pedicled flap [50]. The LD pedicled flap is widely used and reliable for both immediate and delayed reconstruction, particularly in patients with insufficient abdominal tissue or unsuitable for microvascular surgery [51]. A recent large retrospective study by Zheng et al. demonstrated its reliability, showing a modest 2.9% complication rate requiring surgical intervention [52]. However, the LD pedicled flap involves muscle harvest, which may lead to donor site morbidity, and often requires implants to obtain the desired volume [50,53,54]. In our study, only DIEP flaps were included to ensure comparability and reduce heterogeneity, although all other above-mentioned autologous breast reconstructions are performed at our center based on careful patient selection and patient preferences.

Several biases of the present study must be discussed. Our broad inclusion criteria may have contributed to the statistically significant differences observed between groups regarding immediate versus delayed reconstruction, as we excluded all flaps that received postoperative RT and/or CT—generally immediate reconstruction cases.

We also included both unilateral and bilateral flaps, which introduced a major bias in calculating mean operative time, as it was calculated on a per-operation basis. However, when bilateral reconstructions were excluded, operative times were comparable across groups. This factor did not affect ischemia time analysis, as it was calculated on a per-flap basis. The proportion of prophylactic mastectomies being the highest in the CT group is also attributed to the inclusion of bilateral cases. In fact, all prophylactic mastectomies of our cohort were associated with a contralateral oncologic mastectomy with or without previous treatment history. In this case, eight flaps with previous prophylactic mastectomies were included in a treatment group.

The interval between RT completion and reconstruction is also a topic of interest in the literature. Most studies suggest that the timing of the reconstruction after the completion of RT does not affect overall recipient site complication rates [23,55,56]. There are no studies focusing on this interval between CT and reconstruction. However, our calculation of intervals between the end of therapies (RT or CT) and the DIEP-flap reconstruction may be biased because we included both primary and secondary reconstructions. Specifically, 27 patients had previously received RT after breast-conserving surgery, followed by a mastectomy for a later recurrence, while 41 patients underwent RT after mastectomy prior to delayed reconstruction. In both scenarios, the recipient site had been irradiated before reconstruction; therefore, we decided to attribute these patients to RT or RT + CT groups according to their adjuvant treatments.

Other important limitations and biases of the study must also be acknowledged. The retrospective design carries inherent risks of selection bias and limits causal inferences. The sample size, particularly in the RT and CT groups, may not have been sufficient to detect statistically significant differences.

However, our study has several strengths, including the focused assessment of the combined impact of preoperative RT and CT on DIEP flap reconstruction outcomes. By categorizing patients into four distinct groups, we were able to compare the isolated and combined effects of these treatments. The study population was exclusively composed of DIEP flap breast reconstructions to ensure comparability. Additionally, we reported complications that arose after the initial hospital discharge, with an average follow-up period of 16 months, ensuring a thorough evaluation of late postoperative complications.

Future research should focus on prospective studies with larger cohorts to validate our findings. Investigations into the optimal timing between RT, CT, and reconstruction could provide valuable guidance for clinical decision-making. Additionally, exploring interventions to improve tissue vascularity and wound healing, such as hyperbaric oxygen therapy, may offer potential benefits in this high-risk patient population.

## 5. Conclusions

In conclusion, our study shows that preoperative RT or CT alone did not significantly impact intra- or postoperative outcomes of DIEP flap reconstruction. However, the combination of both RT and CT was associated with a significant increase in microsurgical complications. Ischemia time was identified as an independent risk factor for total recipient site complications, but not for microvascular complications alone. These findings underscore the importance of careful preoperative planning and individualized patient management to optimize surgical outcomes in breast reconstruction following cancer treatment.

## Figures and Tables

**Table 1:** Baseline characteristics of the patient population.

n = 114 Patients	Control Group	RT	CT	RT + CT	*p*-Value
	n = 29	n = 21	n = 17	n = 47	
Age [years], mean (SD)	49.0 (6.3)	54.3 (11.1)	46.0 (9.9)	49.0 (8.7)	0.037
BMI [kg/m^2^], mean (SD)	25.9 (4.6)	26.1 (4.7)	28.3 (3.7)	27.3 (4.2)	0.259
ASA score					
ASA 1, n (%)	6 (20.7%)	1 (4.8%)	5 (29.4%)	6 (12.8%)	0.162
ASA 2, n (%)	23 (79.3%)	20 (95.2%)	12 (70.6%)	41 (87.2%)	
Comorbidities					
Active smoker, n (%)	9 (31.0%)	4 (19.0%)	4 (23.5%)	10 (21.3%)	0.050
Obesity (BMI > 30 kg/m^2^), n (%)	5 (17.2%)	5 (23.4%)	6 (35.3%)	12 (25.5%)	0.588
HTN, n (%)	2 (6.9%)	4 (19.0%)	3 (17.6%)	8 (17.0%)	
Diabetes mellitus, n (%)	1 (3.4%)	3 (14.3%)	1 (5.9%)	2 (4.3%)	0.569
Abdominal surgery history, n (%)	14 (48.3%)	16 (76.2%)	7 (41.1%)	19 (40.4%)	0.060

Continuous variables are reported as mean ± standard deviation. Discrete variables are reported according to their frequencies. BMI: body mass index. ASA: American Society of Anesthesiologists (ASA) score. HTN: hypertension.

**Table 2:** Operative variables per patient and per flap.

n = 114 Patients	Control Group	RT	CT	RT + CT	*p*-Value
	n = 29	n = 21	n = 17	n = 47	
Immediate/Delayed reconstruction					
Immediate, n (%)	12 (41.4%)	5 (23.8%)	7 (41.2%)	7 (14.9%)	0.040
Delayed, n (%)	17 (58.6%)	16 (76.2%)	10 (58.8%)	40 (85.1%)	
Radiotherapy administration					
Post breast-conserving surgery, n (%)	N.A.	10 (47.6%)	N.A.	17 (36.2%)	0.38
Post mastectomy, n (%)	N.A.	11 (52.4%)	N.A.	30 (63.8%)	
Unilateral/Bilateral reconstruction					
Unilateral, n (%)	25 (86.2%)	20 (95.2%)	5 (29.4%)	37 (78.7%)	<0.001
Bilateral, n (%)	4 (13.8%)	1 (4.8%)	12 (70.5%)	10 (21.3%)	
Operation time [minutes], mean (SD)	466 (127.1)	486.6 (133.6)	601 (144.4)	506.9 (107)	0.005
Postoperative length of stay [days], mean (SD)	9 (2.4)	9 (2.6)	10.2 (5.2)	10 (3.5)	0.636
Type of flap n = 141					
DIEP, n (%)	33 (100%)	22 (100%)	29 (100%)	57 (100%)	1.000
Ischemia time [minutes], mean (SD)	100 (31.3)	85 (35.5)	98 (32.7)	94 (35.6)	0.512
Number of flap perforators, mean (SD)	2 (0.8)	2 (0.92)	2 (0.6)	2 (1.1)	0.947
Target vessel					
IMA, n (%)	33 (100%)	22 (100%)	29 (100%)	57 (100%)	1.000
Venous anastomosis with coupler, n (%)	14 (42.4%)	15 (68.2%)	15 (51.7%)	34 (59.6%)	0.233
Surgical indication					
Prophylactic, n (%)	2 (6.1%)	0 (0.0%)	8 (27.5%)	0 (0.0%)	<0.001
Oncologic, n (%)	31 (93.9%)	22 (100%)	21 (72.4%)	57 (100%)	
Type of previous Mastectomy					
SSM, n (%)	23 (69.7%)	16 (72.7%)	15 (51.7%)	33 (57.9%)	0.309
NSM, n (%)	9 (27.3%)	4 (18.2%)	11 (37.9%)	7 (12.3%)	0.043
Simple mastectomy, n (%)	1 (3.0%)	2 (9.1%)	3 (10.4%)	17 (29.8%)	0.004

IMA: Internal mammary artery. SSM: skin-sparing mastectomy, NSM: nipple-sparing mastectomy. N.A.: Not applicable.

**Table 3:** Recipient site complications.

n = 141 DIEPs	Control Group	RT	CT	RT + CT	*p*-Value
*N* of Flaps	n = 33	n = 22	n = 29	n = 57	
Flaps without complications, n (%)	29 (87.9%)	18 (81.8%)	26 (89.7%)	43 (75.4%)	0.306
Flaps with ≥1 complication, n (%)	4 (12.1%)	4 (18.2%)	3 (10.3%)	14 (24.6%)	
Seroma, n (%)	0 (0.0%)	1 (4.5%)	0 (0.0%)	0 (0.0%)	0.141
Hematoma, n (%)	0 (0.0%)	2 (4.5%)	1 (3.4%)	2 (1.8%)	0.333
Wound infection, n (%)	0 (0.0%)	0 (0.0%)	1 (3.4%)	1 (1.8%)	0.638
Wound dehiscence, n (%)	1 (3.0%)	1 (4.5%)	1 (3.4%)	2 (3.5%)	0.993
Delayed wound healing, n (%)	2 (6.1%)	1 (4.5%)	0 (0.0%)	2 (3.5%)	0.627
Partial NAC necrosis, n (%)	0 (0.0%)	1 (4.5%)	0 (0.0%)	1 (1.8%)	0.480
Flaps with ≥1 microvascular complication, n (%)	1 (3.0%)	0 (0.0%)	0 (0.0%)	8 (14.0%)	0.021
Venous congestion, n (%)	1 (3.0%)	0 (0.0%)	0 (0.0%)	0 (0.0%)	0.348
Venous thrombosis, n (%)	0 (0.0%)	0 (0.0%)	0 (0.0%)	2 (3.5%)	0.393
Intraoperative arterial thrombosis, n (%)	0 (0.0%)	0 (0.0%)	0 (0.0%)	1 (1.8%)	0.686
Flap loss, n (%)	0 (0.0%)	0 (0.0%)	0 (0.0%)	2 (3.5%)	0.393
Partial flap loss, n (%)	0 (0.0%)	0 (0.0%)	0 (0.0%)	3 (5.3%)	0.211

**Table 4:** Abdominal (donor site) complications per patient.

n = 114 Patients	Control Group	RT	CT	RT + CT	*p*-Value
	n = 29	n = 21	n = 17	n = 47	
No complication, n (%)	22 (75.9%)	19 (90.5%)	11 (64.7%)	26 (55.3%)	0.025
≥1 complication, n (%)	7 (24.1%)	2 (9.5%)	6 (35.3%)	21 (44.7%)	
Complication types					
Seroma, n (%)	2 (6.9%)	0 (0.0%)	2 (11.8%)	2 (4.3%)	0.414
Hematoma, n (%)	1 (3.4%)	0 (0.0%)	0 (0.0%)	3 (6.4%)	0.470
Wound infection, n (%)	0 (0.0%)	0 (0.0%)	1 (5.9%)	2 (4.3%)	0.469
Wound dehiscence, n (%)	2 (6.9%)	1 (4.8%)	1 (5.9%)	6 (12.8%)	0.645
Delayed wound healing, n (%)	2 (6.9%)	1 (4.8%)	2 (11.8%)	7 (14.9%)	0.547
Incisional hernia, n (%)	0 (0.0%)	0 (0.0%)	0 (0.0%)	1 (2.1%)	0.697

**Table 5:** Final logistic regression model: total recipient site complications as a dependent variable.

	Odds Ratio (OR)	95% Confidence Interval (95%-CI)	*p*-Value
Age	1.023	0.960–1.090	0.475
Smoker	1.696	0.539–5.33	0.366
RT	1.302	0.236–7.191	0.762
CT	0.832	0.162–4.277	0.826
RT + CT	1.607	0.456–5.659	0.460
Operation time	0.999	0.995–1.003	0.717
Ischemia time	1.019	1.004–1.035	0.014

**Table 6:** Final logistic regression model: microvascular recipient site complications as a dependent variable.

	Odds Ratio (OR)	95% Confidence Interval (95%-CI)	*p*-Value
Age	0.941	0.845–1.047	0.262
Smoker	1.529	0.245–9.220	0.643
RT	0.000	0.000–N.A.	0.999
CT	1.309	0.072–23.902	0.856
RT + CT	5.558	0.575–53.751	0.138
Operation time	1.000	0.993–1.006	0.927
Ischemia time	1.014	0.992–1.036	0.207

**Table 7:** Final logistic regression model: donor-site complications as a dependent variable.

	Odds Ratio (OR)	95% Confidence Interval (95%-CI)	*p*-Value
Age	1.008	0.961–1.056	0.752
BMI	1.001	0.908–1.102	0.988
Smoker	1.698	0.608–4.238	0.257
CT	0.814	0.266–2.492	0.718
Operation time	0.998	0.995–1.001	0.222
Abdominal surgery history	1.521	0.679–3.407	0.308

## Data Availability

The data presented in this study are available on request from the corresponding author. (The data are not publicly available due to ethical restrictions.).

## Abbreviations

The following abbreviations are used in this manuscript:

RT	Radiotherapy
CT	Chemotherapy
DIEP	Deep inferior epigastric perforator
BMI	Body Mass Index
HTN	Hypertension
ASA	Anesthesiologists
SD	Standard Deviation
Gy	Grey
NAC	Nipple-areolar-complex
OR	Odds Ratio
HBOT	Hyperbaric oxygen therapy

## References

[1] Bray F., Laversanne M., Sung H., Ferlay J., Siegel R.L., Soerjomataram I., Jemal A. (2024). Global cancer statistics 2022: GLOBOCAN estimates of incidence and mortality worldwide for 36 cancers in 185 countries. CA Cancer J. Clin..

[2] Mégevand V., Scampa M., McEvoy H., Kalbermatten D.F., Oranges C.M. (2022). Comparison of Outcomes Following Prepectoral and Subpectoral Implants for Breast Reconstruction: Systematic Review and Meta-Analysis. Cancers.

[3] Recht A., Comen E.A., Fine R.E., Fleming G.F., Hardenbergh P.H., Ho A.Y., Hudis C.A., Hwang E.S., Kirshner J.J., Morrow M. (2017). Postmastectomy Radiotherapy: An American Society of Clinical Oncology, American Society for Radiation Oncology, and Society of Surgical Oncology Focused Guideline Update. Ann. Surg. Oncol..

[4] Taghizadeh R., Moustaki M., Harris S., Roblin P., Farhadi J. (2015). Does post-mastectomy radiotherapy affect the outcome and prevalence of complications in immediate DIEP breast reconstruction? A prospective cohort study. J. Plast. Reconstr. Aesthet. Surg..

[5] Polgár C., Fodor J., Major T., Takácsi-Nagy Z., Kásler M., Hammer J., Van Limbergen E., Németh G. (2002). Radiotherapy confined to the tumor bed following breast conserving surgery current status, controversies, and future projects. Strahlenther. Onkol..

[6] Martineau J., Tekdogan B., Lam G.-T., Correia D., Giordano S., Kalbermatten D.F., Oranges C.M. (2024). Oncological and Surgical Outcomes of Oncoplastic Reduction Mammoplasty: A Single-centre Retrospective Study. In Vivo.

[7] Naoum G.E., Oladeru O.T., Niemierko A., Salama L., Winograd J., Colwell A., Arafat W.O., Smith B., Ho A., Taghian A.G. (2020). Optimal breast reconstruction type for patients treated with neoadjuvant chemotherapy, mastectomy followed by radiation therapy. Breast Cancer Res. Treat..

[8] Cortazar P., Zhang L., Untch M., Mehta K., Costantino J.P., Wolmark N., Bonnefoi H., Cameron D., Gianni L., Valagussa P. (2014). Pathological complete response and long-term clinical benefit in breast cancer: The CTNeoBC pooled analysis. Lancet.

[9] Killelea B.K., Yang V.Q., Mougalian S., Horowitz N.R., Pusztai L., Chagpar A.B., Lannin D.R. (2015). Neoadjuvant chemotherapy for breast cancer increases the rate of breast conservation: Results from the National Cancer Database. J. Am. Coll. Surg..

[10] Anampa J., Makower D., Sparano J.A. (2015). Progress in adjuvant chemotherapy for breast cancer: An overview. BMC Med..

[11] Damen T.H., Timman R., Kunst E.H., Gopie J.P., Bresser P.J., Seynaeve C., Menke-Pluijmers M.B., Mureau M.A., Hofer S.O., Tibben A. (2010). High satisfaction rates in women after DIEP flap breast reconstruction. J. Plast. Reconstr. Aesthet. Surg..

[12] Martineau J., Kalbermatten D.F., Oranges C.M. (2022). Safety and Efficacy of the Superior Gluteal Artery Perforator (SGAP) Flap in Autologous Breast Reconstruction: Systematic Review and Meta-Analysis. Cancers.

[13] Martineau J., Scampa M., Viscardi J.A., Giordano S., Kalbermatten D.F., Oranges C.M. (2023). Inferior gluteal artery perforator (IGAP) flap in autologous breast reconstruction: A proportional meta-analysis of surgical outcomes. J. Plast. Reconstr. Aesthet. Surg..

[14] Tekdogan B., Martineau J., Kalbermatten D.F., Oranges C.M. (2024). Unilateral Versus Bilateral Deep Inferior Epigastric Perforator Flap Breast Reconstruction: A Systematic Review and Meta-analysis. Plast. Reconstr. Surg. Glob. Open.

[15] Garza R.M., Paik K.J., Chung M.T., Duscher D., Gurtner G.C., Longaker M.T., Wan D.C. (2014). Studies in fat grafting: Part III. Fat grafting irradiated tissue--improved skin quality and decreased fat graft retention. Plast. Reconstr. Surg..

[16] Ho A.Y., Hu Z.I., Mehrara B.J., Wilkins E.G. (2017). Radiotherapy in the setting of breast reconstruction: Types, techniques, and timing. Lancet Oncol..

[17] Anavekar N.S., Rozen W.M., Le Roux C.M., Ashton M.W. (2011). Achieving autologous breast reconstruction for breast cancer patients in the setting of post-mastectomy radiotherapy. J. Cancer Surviv..

[18] Sekiguchi K., Kawamori J., Yamauchi H. (2017). Breast reconstruction and postmastectomy radiotherapy: Complications by type and timing and other problems in radiation oncology. Breast Cancer.

[19] Varghese J., Gohari S.S., Rizki H., Faheem M., Langridge B., Kümmel S., Johnson L., Schmid P. (2021). A systematic review and meta-analysis on the effect of neoadjuvant chemotherapy on complications following immediate breast reconstruction. Breast.

[20] Sabitovic A., Trøstrup H., Damsgaard T.E. (2023). The impact of neoadjuvant chemotherapy on surgical outcomes following autologous and implant-based immediate breast reconstruction: A systematic review and meta-analysis. J. Plast. Reconstr. Aesthet. Surg..

[21] Fertsch S., Munder B., Andree C., Witzel C., Stambera P., Schulz T., Hagouan M., Gruter L., Aufmesser B., Staemmler K. (2021). Risk Factor Analysis for Flap and Donor Site Related Complications in 1274 DIEP Flaps—Retrospective Single Center Study. Chirurgia.

[22] Kelley B.P., Ahmed R., Kidwell K.M., Kozlow J.H., Chung K.C., Momoh A.O. (2014). A systematic review of morbidity associated with autologous breast reconstruction before and after exposure to radiotherapy: Are current practices ideal?. Ann. Surg. Oncol..

[23] Arnautovic A., Karinja S., Olafsson S., Carty M.J., Erdmann-Sager J., Caterson S.A., Broyles J.M. (2023). Optimal Timing of Delayed Microvascular Breast Reconstruction after Radiation Therapy. J. Reconstr. Microsurg..

[24] Chi W., Zhang Q., Li L., Chen M., Xiu B., Yang B., Wu J. (2023). Immediate Breast Reconstruction After Neoadjuvant Chemotherapy: Factors Associated With Surgical Selection and Complications. Ann. Plast. Surg..

[25] Zinner G., Martineau J., Lam G.-T., Tremp M., Giordano S., Dong E.T.C., Kalbermatten D.F., Oranges C.M. (2024). Does prepectoral placement delay adjuvant therapies compared to retropectoral immediate implant-based breast reconstruction? A retrospective analysis. J. Plast. Reconstr. Aesthetic Surg..

[26] Alves A.S., Tan V., Scampa M., Kalbermatten D.F., Oranges C.M. (2022). Complications of Immediate versus Delayed DIEP Reconstruction: A Meta-Analysis of Comparative Studies. Cancers.

[27] Ward J., Ho K., Ike C., Wood S.H., Thiruchelvam P.T.R., Khan A.A., Leff D.R. (2024). Pre-operative chemoradiotherapy followed by mastectomy and breast reconstruction-A systematic review of clinical, oncological, reconstructive and aesthetic outcomes. J. Plast. Reconstr. Aesthet. Surg..

[28] Thiruchelvam P.T.R., Leff D.R., Godden A.R., Cleator S., Wood S.H., Kirby A.M., Jallali N., Somaiah N., Hunter J.E., Henry F.P. (2022). Primary radiotherapy and deep inferior epigastric perforator flap reconstruction for patients with breast cancer (PRADA): A multicentre, prospective, non-randomised, feasibility study. Lancet Oncol..

[29] Miyazawa K., Satake T., Muto M., Tsunoda Y., Koike T., Narui K., Katsuragi R., Onoda S., Ishikawa T. (2024). Delayed breast reconstruction with autologous free flap after radiation therapy: Vascular complications and aesthetic outcomes. Breast Cancer.

[30] Khajuria A., Charles W.N., Prokopenko M., Beswick A., Pusic A.L., Mosahebi A., Dodwell D.J., Winters Z.E. (2020). Immediate and delayed autologous abdominal microvascular flap breast reconstruction in patients receiving adjuvant, neoadjuvant or no radiotherapy: A meta-analysis of clinical and quality-of-life outcomes. BJS Open.

[31] Greco J.A., Castaldo E.T., Nanney L.B., Wu Y.C., Donahue R., Wendel J.J., Hagan K.F., Shack R.B. (2008). Autologous breast reconstruction: The Vanderbilt experience (1998 to 2005) of independent predictors of displeasing outcomes. J. Am. Coll. Surg..

[32] Fosnot J., Fischer J.P., Smartt J.M., Low D.W., Kovach S.J., Wu L.C., Serletti J.M. (2011). Does previous chest wall irradiation increase vascular complications in free autologous breast reconstruction?. Plast. Reconstr. Surg..

[33] Fracol M.E., Basta M.N., Nelson J.A., Fischer J.P., Wu L.C., Serletti J.M., Fosnot J. (2016). Bilateral Free Flap Breast Reconstruction After Unilateral Radiation: Comparing Intraoperative Vascular Complications and Postoperative Outcomes in Radiated Versus Nonradiated Breasts. Ann. Plast. Surg..

[34] Schaverien M.V., Munnoch D.A. (2013). Effect of neoadjuvant chemotherapy on outcomes of immediate free autologous breast reconstruction. Eur. J. Surg. Oncol..

[35] Riba J., de Romani S.E., Masia J. (2018). Neoadjuvant Chemotherapy for Breast Cancer Treatment and the Evidence-Based Interaction with Immediate Autologous and Implant-Based Breast Reconstruction. Clin. Plast. Surg..

[36] Warren Peled A., Itakura K., Foster R.D., Hamolsky D., Tanaka J., Ewing C., Alvarado M., Esserman L.J., Hwang E.S. (2010). Impact of chemotherapy on postoperative complications after mastectomy and immediate breast reconstruction. Arch. Surg..

[37] Nag S., Berlin L., Hunter K., Bonawitz S.C. (2024). Effects of Neoadjuvant Chemotherapy on Autologous and Implant-Based Breast Reconstruction: A Systematic Review and Meta-Analysis of the Literature. Clin. Breast Cancer.

[38] Beugels J., Meijvogel J.L.W., Tuinder S.M.H., Tjan-Heijnen V.C.G., Heuts E.M., Piatkowski A., van der Hulst R. (2019). The influence of neoadjuvant chemotherapy on complications of immediate DIEP flap breast reconstructions. Breast Cancer Res. Treat..

[39] Słonimska P., Sachadyn P., Zieliński J., Skrzypski M., Pikuła M. (2024). Chemotherapy-Mediated Complications of Wound Healing: An Understudied Side Effect. Adv. Wound Care.

[40] Arellano J.A., Comerci A.J., Liu H.Y., Alessandri Bonetti M., Nguyen V.T., Parent B., Bailey E.A., Moreira A.A., Gimbel M.L., Egro F.M. (2024). Complications in Prolonged Intraoperative Ischemia Time in Free Flap Breast Reconstruction: A Systematic Review and Meta-Analysis. Aesthetic Plast. Surg..

[41] Marre D., Hontanilla B. (2013). Increments in ischaemia time induces microvascular complications in the DIEP flap for breast reconstruction. J. Plast. Reconstr. Aesthet. Surg..

[42] Lee K.T., Lee J.E., Nam S.J., Mun G.H. (2013). Ischaemic time and fat necrosis in breast reconstruction with a free deep inferior epigastric perforator flap. J. Plast. Reconstr. Aesthet. Surg..

[43] Grace P.A. (1994). Ischaemia-reperfusion injury. Br. J. Surg..

[44] van den Heuvel M.G.W., Buurman W.A., Bast A., van der Hulst R.R.W.J. (2009). Review: Ischaemia–reperfusion injury in flap surgery. J. Plast. Reconstr. Aesthetic Surg..

[45] Scampa M., Martineau J., Boet S., Pignel R., Kalbermatten D.F., Oranges C.M. (2024). Hyperbaric oxygen therapy outcomes in post-irradiated patient undergoing microvascular breast reconstruction: A preliminary retrospective comparative study. JPRAS Open.

[46] Lee B.T., Agarwal J.P., Ascherman J.A., Caterson S.A., Gray D.D., Hollenbeck S.T., Khan S.A., Loeding L.D., Mahabir R.C., Miller A.S. (2017). Evidence-Based Clinical Practice Guideline: Autologous Breast Reconstruction with DIEP or Pedicled TRAM Abdominal Flaps. Plast. Reconstr. Surg..

[47] Hofer S.O.P., Damen T.H.C., Mureau M.A.M., Rakhorst H.A., Roche N.A. (2007). A Critical Review of Perioperative Complications in 175 Free Deep Inferior Epigastric Perforator Flap Breast Reconstructions. Ann. Plast. Surg..

[48] Macadam S.A., Zhong T., Weichman K., Papsdorf M., Lennox P.A., Hazen A., Matros E., Disa J., Mehrara B., Pusic A.L. (2016). Quality of Life and Patient-Reported Outcomes in Breast Cancer Survivors: A Multicenter Comparison of Four Abdominally Based Autologous Reconstruction Methods. Plast. Reconstr. Surg..

[49] Erdmann-Sager J., Wilkins E.G., Pusic A.L., Qi J., Hamill J.B., Kim H.M., Guldbrandsen G.E., Chun Y.S. (2018). Complications and Patient-Reported Outcomes after Abdominally Based Breast Reconstruction: Results of the Mastectomy Reconstruction Outcomes Consortium Study. Plast. Reconstr. Surg..

[50] Nahabedian M.Y., Patel K. (2016). Autologous flap breast reconstruction: Surgical algorithm and patient selection. J. Surg. Oncol..

[51] Bostwick J., Scheflan M. (1980). The latissimus dorsi musculocutaneous flap: A one-stage breast reconstruction. Clin. Plast. Surg..

[52] Zheng S., Hao S., Chen J., Zhang Y., Yang B., Huang X., Liu G., Shao Z., Wu J. (2023). Latissimus dorsi flap—The main force in breast reconstruction for breast tumor in Chinese population. Front. Oncol..

[53] Saldanha I.J., Cao W., Broyles J.M., Adam G.P., Bhuma M.R., Mehta S., Dominici L.S., Pusic A.L., Balk E.M. (2021). AHRQ Comparative Effectiveness Reviews. Breast Reconstruction After Mastectomy: A Systematic Review and Meta-Analysis.

[54] Steffenssen M.C.W., Kristiansen A.H., Damsgaard T.E. (2019). A Systematic Review and Meta-analysis of Functional Shoulder Impairment After Latissimus Dorsi Breast Reconstruction. Ann. Plast. Surg..

[55] Mull A.B., Qureshi A.A., Zubovic E., Rao Y.J., Zoberi I., Sharma K., Myckatyn T.M. (2017). Impact of Time Interval between Radiation and Free Autologous Breast Reconstruction. J. Reconstr. Microsurg..

[56] Mirza H.N., Berlin N.L., Sugg K.B., Chen J.S., Chung K.C., Momoh A.O. (2024). The Impact of Timing of Delayed Autologous Breast Reconstruction following Postmastectomy Radiation Therapy on Postoperative Morbidity. J. Reconstr. Microsurg..

